# Development and Testing of an Ultrasound-Compatible Cardiac Phantom for Interventional Procedure Simulation Using Direct Three-Dimensional Printing

**DOI:** 10.1089/3dp.2019.0097

**Published:** 2020-12-16

**Authors:** Shu Wang, Yohan Noh, Jemma Brown, Sébastien Roujol, Ye Li, Shuangyi Wang, Richard Housden, Mar Casajuana Ester, Maleha Al-Hamadani, Ronak Rajani, Kawal Rhode

**Affiliations:** ^1^ School of Biomedical Engineering & Imaging Sciences, St Thomas' Hospital, King's College London, London, United Kingdom.; ^2^ British Heart Foundation Centre, St Thomas' Hospital, King's College London, London, United Kingdom.

**Keywords:** cardiac phantom, interventional cardiology, 3D printing, multimodal imaging

## Abstract

Organ phantoms are widely used for evaluating medical technologies, training clinical practitioners, as well as surgical planning. In the context of cardiovascular disease, a patient-specific cardiac phantom can play an important role for interventional cardiology procedures. However, phantoms with complicated structures are difficult to fabricate by conventional manufacturing methods. The emergence of three-dimensional (3D) printing with soft materials provides the opportunity to produce phantoms with complex geometries and realistic properties. In this work, the aim was to explore the use of a direct 3D printing technique to produce multimodal imaging cardiac phantoms and to test the physical properties of the new materials used, namely the Poro-Lay series and TangoPlus. The cardiac phantoms were first modeled using real data segmented from a patient chest computer tomography (CT) scan and then printed with the novel materials. They were then tested quantitatively in terms of stiffness and ultrasound (US) acoustic values and qualitatively with US, CT, and magnetic resonance imaging systems. From the stiffness measurements, Lay-fomm 40 had the closest Young's modulus to real myocardium with an error of 29–54%, while TangoPlus had the largest difference. From the US acoustics measurements, Lay-fomm 40 also demonstrated the closest soft tissue-mimicking properties with both the smallest attenuation and impedance differences. Furthermore, the imaging results show that the phantoms are compatible with multiple imaging modalities and thus have potential to be used for interventional procedure simulation and testing of novel interventional devices. In conclusion, direct 3D printing with Poro-Lay and TangoPlus is a promising method for manufacture of multimodal imaging phantoms with complicated structures, especially for soft patient-specific phantoms.


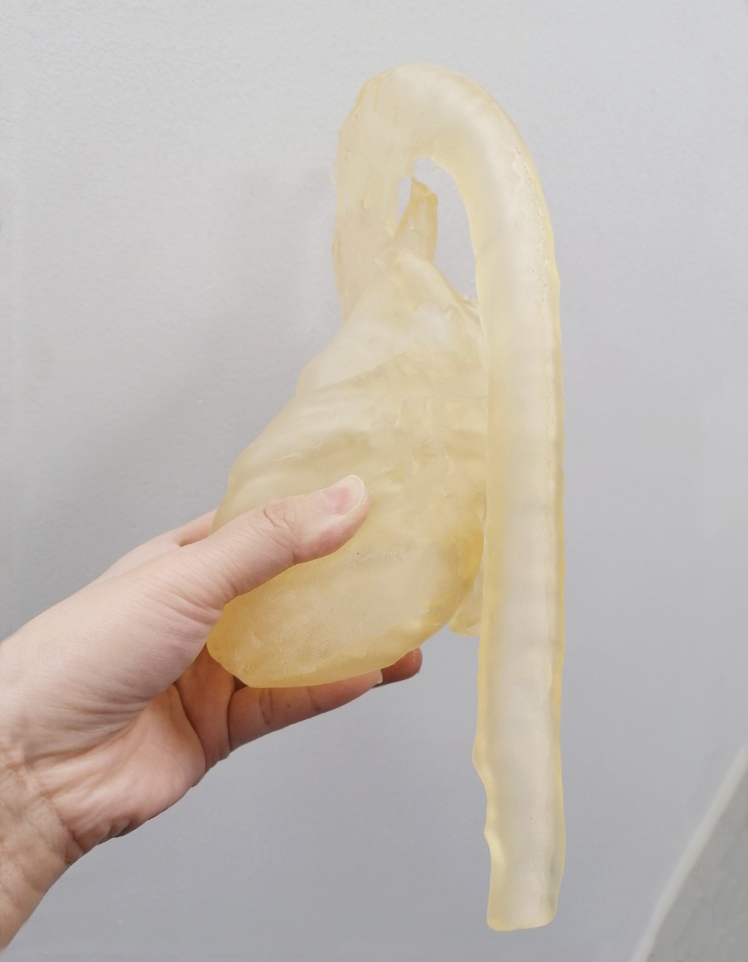


## Introduction

Organ phantoms (anatomical models) are routinely used for development and validation of medical devices, medical education, surgical training, and surgical procedure rehearsal. They reduce the dependency on using animals or cadavers, which are expensive and ethically challenging alternatives. Furthermore, the ability to make phantoms that are specific to a particular patient via medical images allows rehearsal of complex surgical procedures and training of surgeons for a range of patient anatomies.

In modern medicine, many devices and procedures are designed to be operated in a medical imaging environment, so making anatomical models that are compatible with the most common imaging modalities is a requirement.^1^ To satisfy different medical imaging modalities, a range of soft phantom materials has been thoroughly investigated, such as agarose, gelatin, magnesium silicate, oil, open cell foam, polyvinyl alcohol, condensed milk, water, polyacrylamide gel, polyurethane, tofu, urethane rubber, and Zerdine.^1^ While the desired properties of these materials have already been demonstrated, developing phantoms with complex geometries is still challenging with conventional shaping methods, such as using hard negative molds or liquid perfusion because it will involve significant work of removing the molds.

Therefore, existing cardiac phantoms are mainly available as commercial products with complicated manufacturing and postprocessing methods. Examples include the anthropomorphic phantom used to measure surface radiation exposure,^2^ the fetal cardiac ventricular phantom in four-dimensional sonography,^3^ and the multipurpose multi-tissue ultrasound (US) phantom used for fast cardiac imaging.^4^ Although these phantoms can produce good images of the heart itself, they are usually expensive, noncustomizable, and lacking several functionalities when used for specific imaging purposes.

With the development in recent years of three-dimensional (3D) printing technologies, production of soft phantoms with complex geometries using direct 3D printing has become feasible and could potentially be easier and cheaper for phantom fabrication compared with conventional manufacturing methods. Systematic reviews on the use of 3D printing for the manufacture of imaging phantoms confirm that this is an effective low-cost alternative to traditionally manufactured phantoms.^5–7^

However, the vast majority of the articles reviewed use of rigid materials and only demonstrate compatibility with computer tomography (CT). There is significant scope to explore more recently available soft materials for particular applications and other imaging modalities, such as US. Recently, a complete review was carried out on the use of 3D printing technology for making cardiac phantoms.^8^ However, the cardiac phantoms demonstrated in this article are either subparts, such as parts of the aorta (AO) only, or made of clear rigid materials. The review also points to the future of cardiac 3D printing, in which it is suggested that more accurate and complete 3D soft cardiac phantoms are needed for disease analysis and intervention simulation. To design a phantom for real clinical use, a 3D printed multimaterial cardiac phantom for transcatheter native mitral valve (MV) replacement was presented,^9^ but this phantom fabrication procedure involved different technologies for different anatomies, which is complicated to reproduce.

When a phantom is to be imaged, it is necessary to use a material with appropriate properties for the imaging modality. US imaging in particular requires good soft tissue-mimicking properties^10^ such as low acoustic attenuation and low surface reflection, which depend on the US velocity and bulk modulus.^11^ Material stiffness should thus be a key factor to consider when choosing a material for an US phantom.

Focusing on 3D-printed cardiac phantoms used for US imaging purposes, a partial cardiac phantom including cardiac atrial structures made by a metal mold was recently developed.^12^ The phantom was mainly designed for surgical application with an US-compatible material, Gel-Wax, and was proven to have low acoustic attenuation and reflection. However, during manufacturing, Gel-Wax was found to be too flexible to be directly 3D printed, so the phantom could only be fabricated by liquid perfusion and thus lacked detailed cardiac inner structures.

The soft tissue-mimicking materials generally used for US phantoms can be classified into 14 different categories^1^: agarose-based,^13^ gelatin-based,^14^ magnesium silicate-based, oil-based,^15^ water-based,^14^ open cell foam-based, polyacrylamide gel-based, polyurethane, polyvinyl alcohol-based,^16^ tofu, condensed milk-based, urethane rubber, Zerdine, and polyvinyl chloride plastisol.^17^ All these materials have been tested to have similar or even lower attenuation and reflection values compared with cardiac tissue,^1^ but none of them can be 3D printed directly. As for flexible 3D-printable materials, Przybytek *et al.* demonstrated a complete list of commercial filaments, including rubber-like thermoplastic elastomers and thermoplastic polyurethane.^18^ In testing, these were found to be incompatible with US imaging due to strong reflection and large attenuation.

In summary, the currently available patient-specific cardiac phantoms are known to be useful in clinical applications, but they are either too difficult to manufacture or incomplete without fine geometries, which limits their applications in high-fidelity presurgery planning. Existing materials either have inappropriate properties for US imaging or are not suitable for 3D printing.

The aim to this work was to seek a solution for the production of a complete cardiac phantom for US imaging that has ease of manufacture and is customizable and inexpensive. Direct 3D printing technology was investigated with both a high-end PolyJet printer using TangoPlus material (Stratasys Objet500, Israel) and a commonly used fused deposition modeling (FDM) printer (WASP Delta 2040, Italy) using Poro-Lay material (Kai Parthy, Germany). For 3D-printable materials with good soft tissue-mimicking properties that are potentially compatible for US, Young's modulus, acoustic impedance, attenuation, and backscattering properties of the materials need to be taken into consideration for selection. While the literature has suggested the potential of these new materials to be used for soft phantom fabrication, a complete cardiac phantom directly made by 3D printing has not been reported and the imaging-related physical properties of these materials after 3D printing still remain unknown.

This work demonstrates the fabrication of an US-compatible cardiac phantom using direct 3D printing with novel 3D-printable materials. Results are presented for both quantitative evaluations of the phantoms, including stiffness and US acoustic properties, and qualitative comparison of US, magnetic resonance imaging (MRI), and CT images.

## Materials and Methods

### Stiffness and hardness test

To evaluate the stiffness of the chosen new materials, Young's modulus and Shore A hardness were measured and compared with that of three types of room-temperature-vulcanizing (RTV)-silicones, which are already known to be soft tissue-mimicking materials. The prepared samples were as follows: the Poro-Lay series including Lay-fomm 40, Lay-fomm 60, and Gel-Lay; TangoPlus; and RTV-silicones including Jehbco silicone, silicone-0020, and silicone-0050.

Young's modulus to describe the materials' elasticity was measured using an Instron 3343 tension test machine (Instron, United Kingdom) on samples prepared with 4 mm thickness and 10 mm width and a force range of 0–100 N. For each material, the test was repeated 20 times to reduce random errors. During the tests, the full data set was recorded and exported by the test device's software Bluehills (Instron) and further analyzed in MATLAB 2017 (MathWorks, United Kingdom). Additionally, another measurement using a Shore Type A durometer tester (Gain Express, United Kingdom) was performed for indentation hardness. The samples were prepared with 5 cm thickness, and the data were recorded from the tester's digital screen.

### US parameter test

US properties of the new materials, including attenuation, velocity, acoustic impedance, and backscattering coefficients were measured to evaluate the materials' US imaging compatibility. As different materials have different attenuations for US waves, each sample was prepared at a different thickness with a flat smooth surface to ensure that its attenuation could be measured. The samples were placed on a steel plate in a water bath and imaged 100 times using the pulse-echo method.^13^ An LA332E-2 probe operating at 1.5 MHz was used with the Ultrasound Advanced Open Platform (ULA-OP64-2).^19^ The plate was then imaged again without the sample.

The property values were calculated using equations (1)–(4)^13^ using MATLAB 2017 (MathWorks). The velocity, attenuation, and backscattering values were averaged from the 100 repeated acquisitions. The acoustic velocity was calculated using equation (1), where *c_s_* is the acoustic velocity in the new material sample, *c_w_* is the acoustic velocity in degassed water (1447 m/s), and *d* is the sample thickness measured separately with a digital ruler. ΔT is the time shift upon displacement of water with the sample in place, calculated from the time of reflection from the steel plate.

(1) $$\frac{1}{c_{s}}=\frac{1}{c_{w}}-\frac{\Delta T}{2d}.$$

The attenuation was calculated from the log difference between two acquired spectra in equation (2), where α is the attenuation coefficient, $A\left(f\right)$ is the magnitude of the spectrum at the transmitted US frequency 1.5MHz with the sample in place, and $A_{0}\left(f\right)$ is the magnitude of the spectrum with no sample in place:

(2) $$\alpha =-\frac{20}{2d}log_{10}\frac{A\left(f\right)}{A_{0}\left(f\right)}\left[\frac{dB}{cm}\right].$$

Backscatter coefficients *u* of each sample were measured in dB for the backscatter from the sample relative to the signal from a flat steel reflector. By measuring the reflected pulse from the material surface and the reflected pulse from the metal plate without the sample in place, *u* was calculated using equation (3):

(3) $$u=-20log_{10}\frac{A\left(f\right)}{A_{0}\left(f\right)}\left[dB\right].$$

With velocity measurements, the acoustic impedance was calculated from Z = cρ, where c is the mean velocity transmitted in the sample, as calculated in equation (1). ρ is the material's density calculated from the measured mass and the volume known from the 3D printing model.^20^ Some other derived acoustic values were calculated by using equation (4): the amplitude reflection coefficient r,the amplitude transmission coefficient *t*, the intensity reflection coefficient *R*, and the intensity transmission coefficient *T*, where we take water as having the reference impedance Z_1_ (1.48$\times 10^{6}$kg/$m^{2}∕s$).

(4) $$r=\frac{Z_{1}-Z_{2}}{Z_{1}+Z_{2}},t=\frac{2Z_{2}}{Z_{1}+Z_{2}},R={\left(\frac{Z_{1}-Z_{2}}{Z_{1}+Z_{2}}\right)}^{2},T=\frac{4Z_{1}Z_{2}}{{\left(Z_{1}+Z_{2}\right)}^{2}}.$$

### Three-dimensional model segmentation and printing

Before printing, the cardiac model was manually segmented using ITK-Snap^21^ from a CT scan of a healthy male volunteer. The scan dimensions were 512 × 549 × 519, with a voxel size of 1 × 1 × 1 $mm^{3}$. The segmentation included the left ventricle (LV), right ventricle (RV), left atrium (LA), right atrium, MV, AO, and interventricular septum.

After rough manual segmentation, Seg3D (CIBC) was used to smooth the original segmentation and correct any internal holes. This was performed by applying a median filter of smoothing level 4. After smoothing, the 3D model of the heart was repaired using MeshLab (MeshLab, Italy) to fill holes on the surface.

Based on the stiffness and US acoustic property results from the previous experiment, Lay-fomm 40 was selected for its best flexibility and lowest attenuation among all the 3D-printable materials. Compared with general rigid filaments, Lay-fomm 40 required stricter printer settings. The material required an extruder printing speed less than 40 mm/s and a temperature of 225°C to prevent nozzle clogging. For better printing quality, the filament was dried out in a filament drier before printing. When printing was performed, the Lay-fomm 40 phantom's support material was removed with scissors and tweezers manually.

Throughout cardiac phantom fabrication, the FDM printer was chosen to print the novel filament series Poro-Lay, with a nozzle size of 0.4 mm. For comparison, a Stratasys Objet500 printer, with printing accuracy of 0.2 mm, was used to print a phantom using TangoPlus material. The general printing steps for printing with TangoPlus were similar, except that the material was prepared in the form of gel instead of filament spool. To remove the support materials after printing, the TangoPlus phantom was immersed in 5% soda water for 2 h. After printing, TangoPlus was stored in air without any postprocessing, whereas Lay-fomm 40 must be rinsed in water for 2 days to reach its maximum flexibility.

### Multimodality imaging comparison

After preparing the two cardiac phantoms, they were imaged using multiple imaging modalities: two-dimensional (2D) US, 2D X-ray, CT (cone-beam), and MRI.

The 2D US images were acquired with an iE33 US machine (Philips, Eindhoven, The Netherlands) and linear array X3-1 transducer. The phantom was fixed to the bottom of a box, which was filled with water. During imaging, an ablation catheter was inserted into the RV of the phantom to simulate an interventional cardiology procedure.^22,23^ The X-ray and CT scans were performed by a Siemens Axiom Artis system (Siemens Healthineers, United Kingdom). As both phantom materials—TangoPlus and Lay-fomm 40—have similar mean densities demonstrated in Table 2, and their densities have a large difference to air, to enhance the X-ray and CT image contrast, they were acquired in air for a clear visualization of both the cardiac inner structure and the catheter. A coronary sinus catheter was inserted into the RV of the TangoPlus phantom and the LV of the Lay-fomm 40 phantom. Finally, the MRI validation scans were performed in a Siemens MAGNETOM Aera 1.5T system (Siemens Healthineers). Three-dimensional T1-weighted and 3D T2-weighted data were acquired using a fast spin echo sequences (voxel size = 1.3 × 1.3 × 1.3 $mm^{3}$). Single-slice T1 and T2 mapping were performed using the Modified Look-Locker inversion recovery [5-(3)-3 MOLLI scheme^24^] sequence and a T2-prepared based sequence, both of them using a balanced steady-state free precession readout^25^ (voxel size = 1.4 × 2.1 × 8 $mm^{3}$). Due to the strong magnetic field in the scanning room, no catheter could be inserted. To better compare the phantoms in a realistic soft tissue environment, both phantoms were imaged in a water tank. The experiments involved in this paper are not related to any human subjects, the data related to human being is all referenced, the phantom is made by 3D printing filaments. The collection of the original patient's cardiac CT images is permitted by Guy's and St Thomas's hospital.

## Results and Discussion

### Stiffness comparison

Young's modulus and durometer test results of different materials are summarized in Table 1. The durometer results are consistent with Young's modulus results. From existing results in the literature, TangoPlus is known to have a Shore A durometer value of 26–28 and a Young's modulus of 0.1–1 MPa.^20^ RTV silicone (excluding Jehbco silicone) is softer with a Young's modulus of 0.01–0.2 MPa. Our results shown in Table 1 for these materials are consistent with these previous findings.

**Table 1.** Stiffness Comparison Between Different Materials

Samples	3D printable				Non-3D printable		
	Lay-fomm 40	Lay-fomm 60	Gel-Lay	TangoPlus	Silicone-0050	Silicone-0020	Jehbco silicone
Durometer (HA)	4	16.5	17	29	5	2.5 (min)	39 (max)
Young's modulus (kPa)	128.5	584.8	1493.7	609.5	96.5	17.8 (min)	2350.0 (max)

3D, three-dimensional.

Table 1 gives the stiffness results of both the new 3D-printable materials (the first four columns) and the classic tissue-mimicking silicones (the next three columns). From Table 1, it is can be observed that silicone-0020 is the softest, whereas the Jehbco silicone is the stiffest. The myocardium's Young's modulus is between 0.18 and 0.28 MPa.^20^ In this case, Lay-fomm 40 is softer but has the closest value to real myocardium with an error of 29–54%, whereas TangoPlus is stiffer with an error of 118–239%. Among the 3D-printable materials, it is clear that Lay-fomm 40 is the most flexible one with the smallest durometer and Young's modulus values. At its maximum flexibility, it is 4.74 times softer than TangoPlus in terms of Young's modulus.

As US acoustic properties are related to stiffness, the above stiffness and hardness results can be used as a reference to explain the following US acoustic performance.

### US acoustic properties

The US acoustic values of different materials are summarized in Table 2, including velocity, attenuation, and backscattering. For each property, the minimum and maximum among all the tested materials are highlighted. As Jehbco silicone has a much larger Young's modulus than other materials and soft tissue, it was not tested further in terms of US properties. Here, it can be observed that silicone-0020, which is the most flexible material, also has the lowest attenuation of 2.5 dB/cm at 1.5 MHz, the largest mean backscattering coefficient of −9.32 dB relative to steel plate, and lowest velocity of 993 m/s at 1.5 MHz. In the literature, it is reported that the classic tissue-mimicking material RTV-silicone has a velocity between 959 and 1113 m/s and attenuation between 0.1 and 5.6 dB/cm measured in the frequency range of 500 kHz–1 MHz^26^; our results are compatible with these reference results.

**Table 2.** Ultrasound Acoustic Property of Different Materials

Samples	3D printable				Non-3D printable	
	Lay-fomm 40	Lay-fomm 60	Gel-Lay	TangoPlus	Silicone-0050	Silicone-0020
Mean density (kg/*m*^3^)	1082	1041	1023 (min)	1100	1363 (max)	1202
Mean attenuation (dB/cm at 1.5 MHz)	6.57	5.95	6.24	29.31 (max)	4.62	3.52 (min)
Mean velocity (m/s at 1.5 MHz)	1468	1544	1581	2039 (max)	1063	1038 (min)
Impedance $\left(10^{6}kg∕m^{2}∕s\right)$	1.59	1.61	1.62	2.24 (max)	1.45	1.25 (min)
Backscattering coefficients (dB at 1.5 MHz)	−22.50	−20.41	−17.86	−32.60 (min)	−18.22	−15.26 (max)

Excluding the non-3D-printable RTV-silicone, all the Poro-Lay series materials have significantly less attenuation compared with TangoPlus, while among them, Lay-fomm 40 has the smallest, in terms of acoustic impedance. However, in terms of backscattering coefficients, the Poro-Lay series is similar to TangoPlus and silicone-0050. The attenuation and acoustic impedance of real cardiac tissue are 0.5 dB/cm and 1.67 × $10^{6}\frac{kg}{m^{2}}∕s$,respectively, at 1 MHz,^19^ then according to the linear dependency of attenuation on frequency, the estimated attenuation of real cardiac tissue is 0.75 dB/cm at1.5 MHz. A comparison between the real tissue and the new materials is summarized in Table 3, where the relative difference is calculated by the absolute difference dividing the value of cardiac tissue. From Table 3, TangoPlus has the largest attenuation difference to myocardium, whereas silicone-0020 has the smallest. Among the 3D-printable materials, Lay-fomm 40 has the most soft tissue-mimicking result with the smallest attenuation and close to the smallest impedance difference.

**Table 3.** Relative Acoustic Value Difference Between Different Tissue-Mimicking Materials and Cardiac Tissue

Samples	3D printable				Non-3D printable	
	Lay-fomm 40	Lay-fomm 60	Gel-Lay	TangoPlus	Silicone-0050	Silicone-0020
Attenuation difference (at 1.5 MHz)	776%	693%	732%	3808% (max)	516%	303% (min)
Impedance difference	4.79%	3.59%	2.99% (min)	34.13% (max)	13.17%	25.15%

As direct silicone printing technology is not yet mature enough, the above US acoustic results demonstrate the good potential of using Lay-fomm 40 to fabricate multimodal cardiac phantoms. By comparing stiffness and US acoustic properties among all the 3D-printable materials, Lay-fomm 40 was chosen because of its best flexibility and smallest acoustic attenuation values, whereas TangoPlus was chosen for a better printing quality and easy stable storage.

### Three-dimensional segmentation and printing

**FIG. 1.**
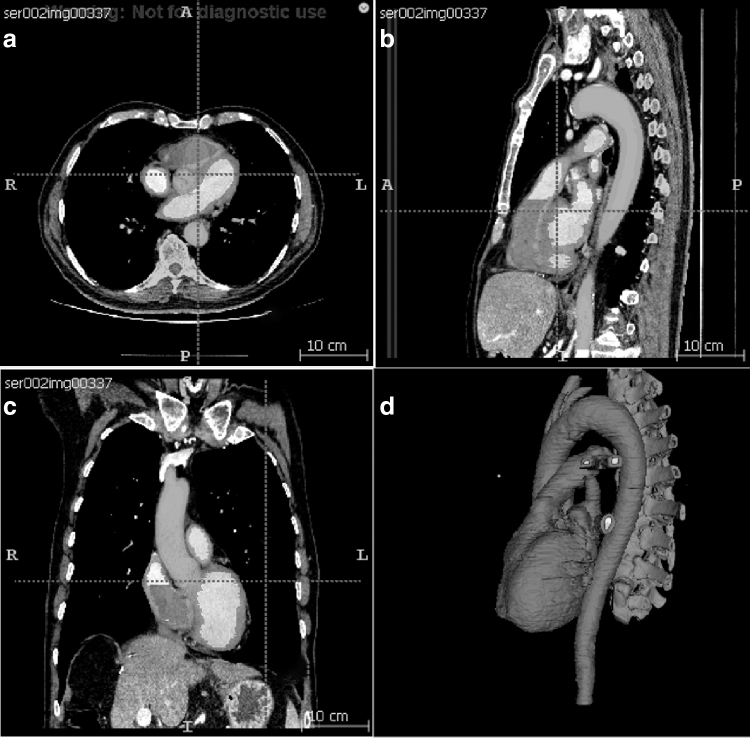
**(a–c)** The cardiac structure and spine segmentation in multiplanar views, and **(d)** the generated 3D model from the three views. 3D, three-dimensional.

**FIG. 2.**
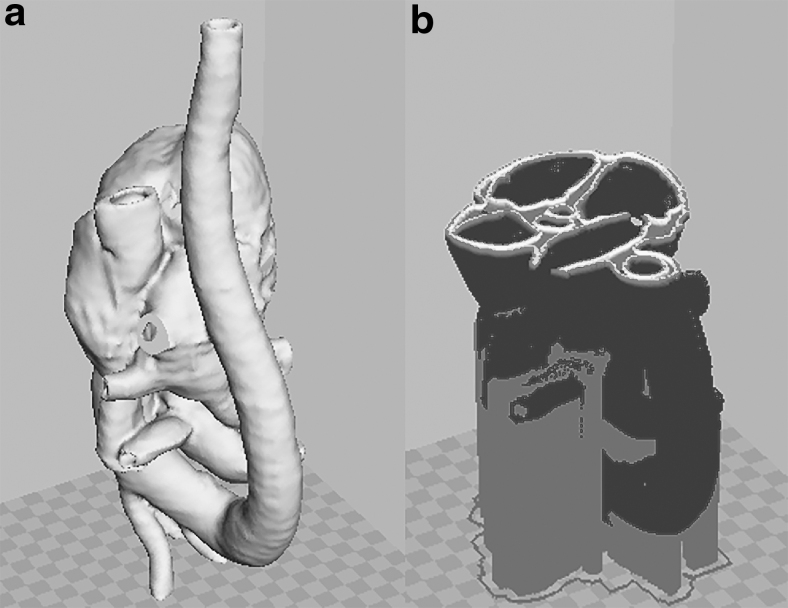
Three-dimensional segmented cardiac model in **(a)** surface view and **(b)** inner slice view, shown in Cura 15.04.2 3D printing software (Ultimaker, The Netherlands).

Figure 1a–c shows the segmentation results in multiplanar views in ITK-SNAP, and Figure 1d shows the initial segmented 3D model. The red label is the myocardium that needs to be printed, whereas the yellow label is the vena cava, the green label is the pulmonary artery, and the brown label is the spine. The red label was exported and postprocessed, producing the 3D-printable cardiac model shown in Figure 2.

It can be observed that the cardiac model generated has a complete structure of four chambers, bicuspid and tricuspid valves, and all the great vessels. The model resembles the real patient heart and could be used in cardiac intervention experiments if adequately printed.

**FIG. 3.**
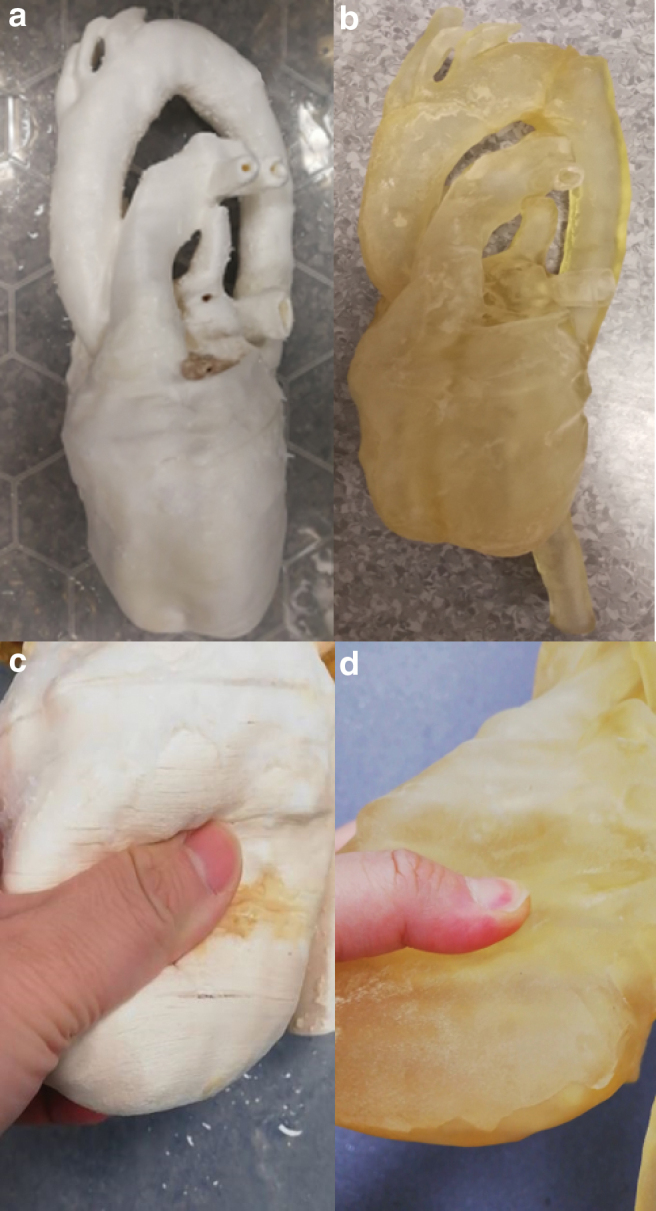
**(a)** Lay-fomm 40 phantom and **(b)** TangoPlus phantom. **(c, d)** Demonstrate the soft properties of these materials using thumb indentation (Supplementary Videos S1 and S2). Color images are available online.

Figure 3 shows the printed results using Lay-fomm 40 and TangoPlus, respectively. This figure also demonstrates ease of indentation using the thumb with the Lay-fomm phantom being much easier to indent than the TangoPlus phantom, which is consistent with the stiffness measurement results presented in Table 1. The overall printed dimensions of both phantoms were 30 × 14 × 11 $cm^{3}$. One of the advantages of 3D printing is that it is easy to scale the final print to suit the application and to balance the costs. For some applications, such as procedure rehearsal, it is important to maintain 100% scaling, but for others, such as testing of imaging systems, less than 100% scaling can be used to reduce overall costs and manufacture time.

We assessed dimensional errors in our phantoms by performing two clinically useful measurements (1) the LV long-axis size as measured from the mid-MV to the cardiac apex and (2) the maximum anteroposterior diameter of the LA. We compared the measurements made on the phantoms with those made on the original patient CT scan. For the Lay-fomm phantom, the measurement errors were (1) 10.5% and (2) 9.4%, and for the TangoPlus phantom, they were (1) 12.7% and (2) 7.9%. The sources of error will include the resolution and quality of the original CT images, the errors in the segmentation of these images, and finally, the errors in the 3D printing process. Our findings indicate an overall error of 10% in dimensions, and this would be acceptable for many of the intended applications. The printing and material cost for the Lay-fomm 40 phantom was 68.70 GBP and for the TangoPlus phantom was 521.24 GBP. In terms of printing requirements, TangoPlus needs a high-end PolyJet printer because it is in the form of liquid gel instead of a normal filament spool, thus making it less affordable. However, once the printing starts, TangoPlus is much easier to control due to its lower melting point. For Lay-fomm 40, which can be printed with any inexpensive desktop FDM printer, the printing may fail due to its higher melting point and stickiness. The resolution of the TangoPlus phantom is 0.2 mm, which is two times higher than the resolution of the Lay-fomm 40 phantom with a resolution of 0.4 mm. Additionally, TangoPlus can be stored in air at room temperature, whereas Lay-fomm 40 can only be stored in water to keep its flexibility.

### Multimodal imaging

**FIG. 4.**
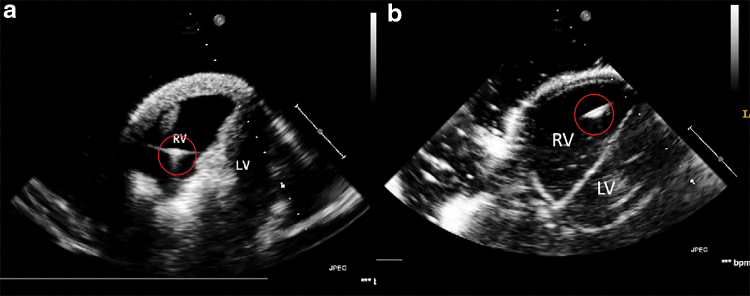
Two-dimensional echocardiography from transthoracic echocardiography parasternal short axis view, with a catheter inserted in the RV labeled with *red circles*. **(a)** Lay-fomm 40 phantom and **(b)** TangoPlus phantom. LV, left ventricle; RV, right ventricle. Color images are available online.

Figure 4 shows the 2D echocardiography of the two cardiac phantoms, during image collection from the X3-1 probe in approximately the parasternal short axis view of transthoracic echocardiography. The ablation catheter was inserted into the RV and appears as the bright spots labeled with the red circles. The resulting 2D echocardiography of the phantoms indicates that both Lay-fomm 40 and TangoPlus are suitable for US phantoms as they both present clear cardiac inner structures.

**FIG. 5.**
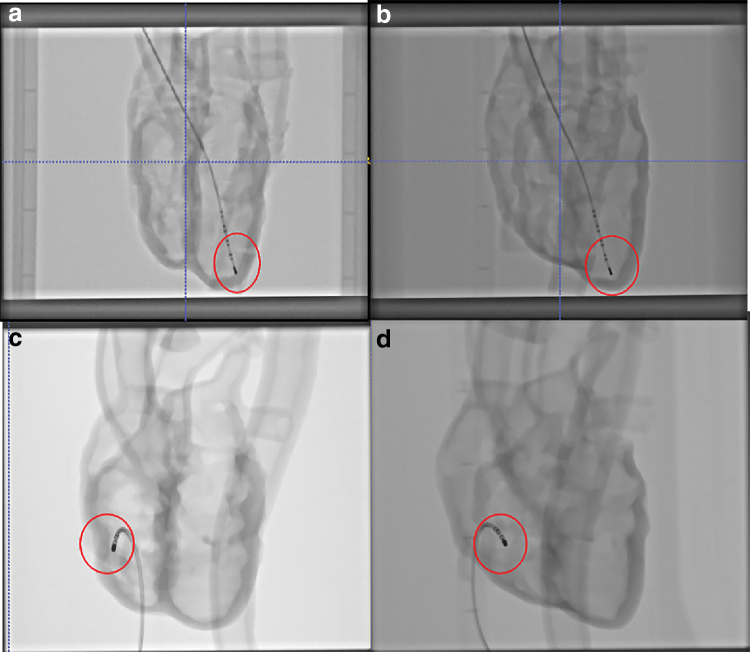
Two-dimensional X-ray images of **(a, b)** Lay-fomm 40 phantom and **(c, d)** TangoPlus phantom from 0° view and 90° view, respectively, with ablation catheters inside the phantom. *Red circles* are the highlights of ablation catheter tips inside different cardiac phantoms. Color images are available online.

**FIG. 6.**
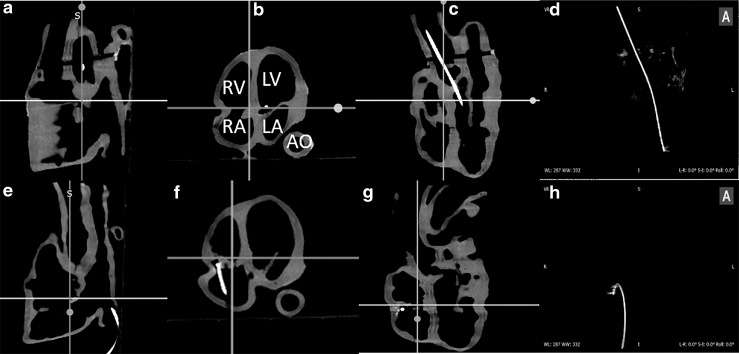
CT images of the cardiac phantoms shown using three multiplanar views: **(a–c)** Lay-fomm 40 phantom and **(e–g)** TangoPlus phantom. **(d**, **h)** Volume renderings of the inserted ablation catheter in the Lay-fomm and TangoPlus phantoms, respectively. AO, aorta; CT, computer tomography; LA, left atrium; RA, right atrium.

**FIG. 7.**
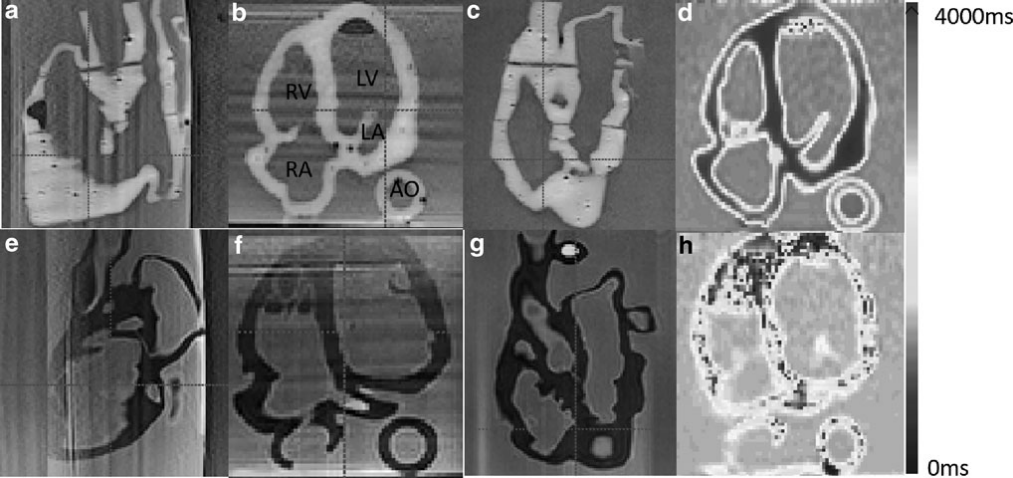
Three-dimensional MRI images of the cardiac phantom using multiplanar views in TI-weighted imaging **(a–c, e–g)** and T1-mapping **(d, h)**. **(a–d)** Lay-fomm 40 phantom and **(e–h)** TangoPlus phantom. MRI, magnetic resonance imaging.

**FIG. 8.**
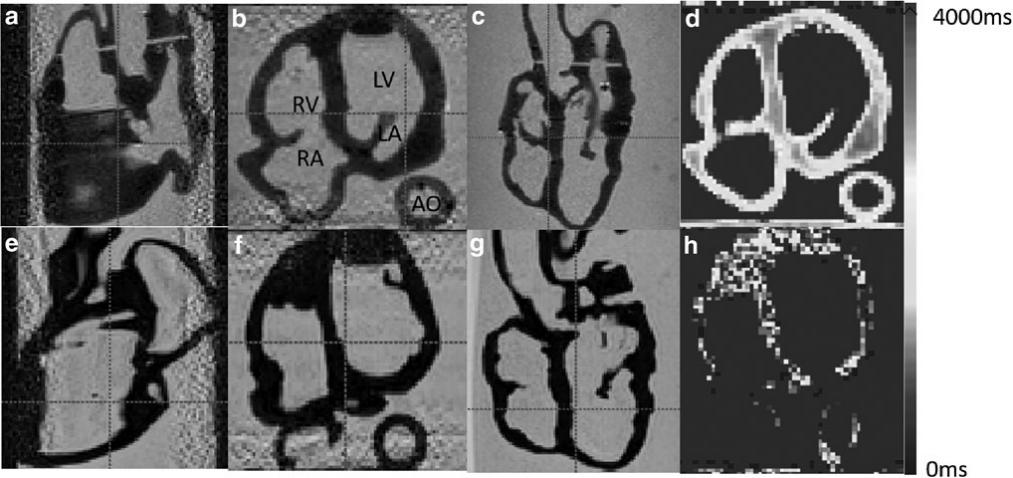
Three-dimensional MRI images of the cardiac phantom using multiplanar views in T2-weighted imaging **(a–c, e–g)** and T2-mapping **(d, h)**. **(a–d)** Lay-fomm 40 phantom and **(e–h)** TangoPlus phantom.

Figures 5 and 6 show the 2D X-ray and 3D CT images of the two phantoms acquired with the Siemens C-arm X-ray machine. The ablation catheter is inserted into the LV of the Lay-fomm 40 heart and the RV of the TangoPlus one. Under X-ray and CT, TangoPlus has no obvious difference to Lay-fomm 40 in terms of pixel intensity. As X-ray and CT images are representations of material densities, the above similarities can be explained by the fact that TangoPlus has a density of 1100 kg/m^3^, which is close to Lay-fomm 40s density of 1082 kg/m^3^. Compared with the echocardiography, we can better track and reconstruct the catheter tip under fluoroscopy, but the cardiac inner structures, especially the valves, are difficult to see in 2D X-ray. Thus, as in the real clinical application, 2D X-ray is especially used for tracking the catheter location, whereas 2D echocardiography is used to guide certain catheter procedures inside the heart. Figures 7 and 8 show the MRI images of the two phantoms without catheters using four imaging sequences: T1-weighted/T1-mapping and T2-weighted/T2 mapping, respectively. The image contrast of Lay fomm 40 phantom is 80%, while TangoPlus is −57% relative to water in T1 mode, and the image contrast of Lay-fomm 40 is −90%, while TangoPlus is −96% relative to water in T2 mode. Over the entire myocardium, T1 and T2 times of the Lay-fomm 40 were 692 and 1267 ms, respectively. While the T1 time of the Lay-fomm was in similar range as *in vivo* myocardial T1 time (∼1000 ms using MOLLI),^21^ its T2 time was larger than *in vivo* myocardial T2 time (∼50 ms).^22^ The reduced quality of T1/T2 maps of the TangoPlus did not allow for a confident quantification of T1/T2 times. This could be explained by the nature of the TangoPlus phantom, which has less water content and is more rigid than the Lay-fomm 40 phantom.

Three-dimensional printing does result in inter- and intra-layer spaces and cavities that could be a potential disadvantage. These were a feature of both of our phantoms. For US imaging, these were advantageous and helped to produce a realistic speckle texture in US images as shown in Figure 4. The effects on CT and MR imaging are minimal, and if we consider the intended use of these phantoms, there is no disadvantage. Delaminations are also a potential problem, and these were experienced with the Lay-fomm phantom over time but not with the TangoPlus phantom, which was prone to fracture over time due to the material becoming brittle.

## Conclusions

This work has explored the use of direct 3D printing technology for cardiac phantom fabrication and presented the results of material property tests on several new soft 3D-printable materials: the Poro-Lay series filaments and TangoPlus. The experimental results for the stiffness and acoustic property tests indicate that the Poro-Lay series, especially Lay-fomm 40, has good mechanical properties to represent soft cardiac tissue quantitatively, whereas TangoPlus can produce better quality printing results for simulation, even though it has larger differences to real cardiac tissue. Thus, they were selected for 3D printing of two complete cardiac phantoms, with the Lay-fomm 40 phantom printed using a common FDM printer and the TangoPlus phantom printed using a high-end PolyJet printer. Both phantoms were also investigated for multimodal imaging compatibility. While no significant differences were observed for 2D X-ray and 3D CT, Lay-fomm 40 showed better performance under US and MRI imaging with fewer artifacts and less boundary reflection in US.

Thus, we conclude that with the investigated new materials, direct 3D printing is feasible for making soft organ phantoms with complex geometry. Two complete cardiac phantoms were successfully fabricated as examples. Additionally, the chosen materials TangoPlus and Lay-fomm 40 demonstrated to be compatible for multimodal imaging, while Lay-fomm 40 produces fewer US artifacts, is visible in other modalities including X-ray and MRI, as well as being cheaper for printing.

## Future Work

The current work focused more on the cardiac phantoms' US performance rather than other imaging modalities, and therefore, further quantitative work will be carried out using X-ray, CT, and MRI. Additionally, there are still certain limitations about soft tissue-mimicking phantom development with 3D printing technology in terms of material choices and fabrication complexity; for example, silicone is a good choice for making tissue-mimicking phantoms, but the molding procedure with silicone is very complicated. In this case, direct silicone printing could become a very interesting and promising direction.

## Supplementary Material

Supplemental data

Supplemental data
